# Staphylococcal internalization into osteoblasts: a partially conserved mechanism across the genus

**DOI:** 10.1128/mbio.01697-25

**Published:** 2025-12-16

**Authors:** Deborah M. Crepin, Mélanie Bonhomme, Allison Faure, Clara Sinel, Marine Bergot, Virginie Dyon-Tafani, Yousef Maali, Daniel Bouvard, Alan Diot, Frédéric Laurent, Jérôme Josse

**Affiliations:** 1 Centre International de Recherche en Infectiologie (CIRI), Team "Pathogénie des Staphylocoques", Inserm U1111, Université Claude Bernard Lyon 1, CNRS UMR 5308, Ecole Normale Supérieure de Lyon, Lyon, France; 2 Centre de Recherche en Biologie Cellulaire (CRBM), Université de Montpellier, CNRS UMR5237 27037, Montpellier, France; 3 Laboratoire de Bactériologie, Institut des Agents Infectieux, Hôpital de la Croix-Rousse, Hospices Civils de Lyon, Lyon, France; 4 Centre National de Référence des Staphylocoques, Institut des Agents Infectieux, Hôpital de la Croix-Rousse, Hospices Civils de Lyon, Lyon, France; University of Texas Health Science Center, School of Public Health, Houston, Texas, USA

**Keywords:** *Staphylococcus *non-*aureus*, fibronectin-binding protein, adhesion, internalization, evolution

## Abstract

*Staphylococcus aureus*, recognized as a major human pathogen, is associated with severe infections such as bacteremia, endocarditis, skin and soft tissue infections, and bone and joint infections. Virulence mechanisms, including biofilm formation and host cell invasion/internalization, contribute to *S. aureus* pathogenicity by allowing it to evade the immune system and most antibiotic treatments. *S. aureus* can be internalized by non-professional phagocytic cells such as fibroblasts, epithelial cells, endothelial cells, and osteoblasts. Its primary internalization pathway is FnBP-fibronectin-α5β1 integrin-dependent. Interestingly, *S. pseudintermedius, S. delphini*, and *S. argenteus* can also invade osteoblasts, relying on FnBP-like proteins such as *S. pseudintermedius* surface proteins D and L (SpsD/L) or *S. delphini* surface protein Y (SdsY). However, the internalization capacity and mechanisms remain poorly explored in other *Staphylococcus* species. Here, we investigated staphylococcal internalization into osteoblasts at the genus level and examined its correlation with FnBP-like proteins, integrating fibronectin adhesion assays, osteoblast infections, and genome analysis. Among the 53 *Staphylococcus* species tested, half exhibited high internalization into osteoblasts. We demonstrated that the FnBP-fibronectin-α5β1 integrin-dependent internalization pathway of *S. aureus* is well conserved in 27 *Staphylococcus* species. *In silico* analyses revealed multiple FnBP-like proteins associated with highly internalized species, showing sequence diversity likely resulting from multiple gene acquisitions throughout *Staphylococcus* evolution.

**IMPORTANCE:**

The internalization of *Staphylococcus aureus* into non-professional phagocytic cells (NPPCs) is considered a key mechanism in the development of persistent infections. The primary internalization pathway has been clearly identified. However, the capacity for internalization and its underlying mechanism remain poorly studied in other *Staphylococcus* species. In this study, we demonstrated that half of the species within the *Staphylococcus* genus are capable of being internalized by osteoblasts using a mechanism similar to that of *S. aureus*. Internalization into NPPCs may therefore represent a partially conserved process within the *Staphylococcus* genus, raising important questions about the evolution of pathogenicity in staphylococci.

## INTRODUCTION

The genus *Staphylococcus* comprises 67 validly published species or subspecies, mostly isolated from human or animal samples (1, 2). Many are commensals of the normal skin and mucosal microbiota but can act as opportunistic pathogens, causing a wide range of infections (3, 4). Among them, *Staphylococcus aureus* is the most common human opportunistic pathogen, responsible for both community and nosocomial infections, including bacteremia, endocarditis, skin and soft tissue infections, and bone and joint infections (BJIs) (5). Other staphylococcal species, such as *S. epidermidis*, *S. capitis*, *S. lugdunensis*, *S. saprophyticus*, and *S. pseudintermedius*, can also cause human infections (6–9).

It is well established that *S. aureus* can be internalized by non-professional phagocytic cells (NPPCs) such as fibroblasts, epithelial cells, endothelial cells, and osteoblasts (10–13). This internalization primarily depends on fibronectin-binding proteins (FnBPA and FnBPB), adhesins located on the bacterial cell wall. FnBPs bind to fibronectin and subsequently to the cellular α5β1 integrin, acting as a bridge between the bacterium and the host cell (14, 15). This interaction remodels the cytoskeleton, inducing cell membrane invagination (16). The FnBPA and FnBPB isoforms, encoded by the *fnbA* and *fnbB* genes (17, 18), belong to the microbial surface components recognizing adhesive matrix molecule (MSCRAMM) family (19).

Data on the internalization of other *Staphylococcus* species into NPPCs remain limited. *S. lugdunensis* can invade epithelial and endothelial cells but not osteoblasts (20–22). *S. epidermidis* can invade osteoblasts, though its internalization does not involve α5β1 integrin (23). Studies by Valour et al. and Campoccia et al. suggest that osteoblast invasion by *S. epidermidis* is significantly lower than that of *S. aureus* (22, 24). Recent research confirms that *S. pseudintermedius, S. delphini,* and *S. argenteus* can invade osteoblasts (25–27), using FnBP-like proteins such as surface proteins D and L (SpsD/L) in *S. pseudintermedius* or SdsY in *S. delphini* (25, 26, 28). However, a comprehensive genus-wide study is lacking.

We investigated the osteoblast internalization capacity across multiple *Staphylococcus* species and its correlation with FnBP-like proteins. Using genomic analysis, fibronectin adhesion assays, and osteoblast invasion quantification, we examined 53 validly published staphylococcal species until 2020, each species being represented by its reference strain. Our study revealed that over half of the species can be internalized into osteoblasts, primarily via the “FnBP-like-fibronectin-α5β1 integrin”-dependent pathway. Genomic evidence indicates multiple independent acquisitions of FnBP-like proteins during *Staphylococcus* evolution, possibly through recurrent exchanges among bacterial lineages rather than solely through host-derived events.

## RESULTS

### *In silico* analysis of *Staphylococcus* reference strains

**Fig 1:**
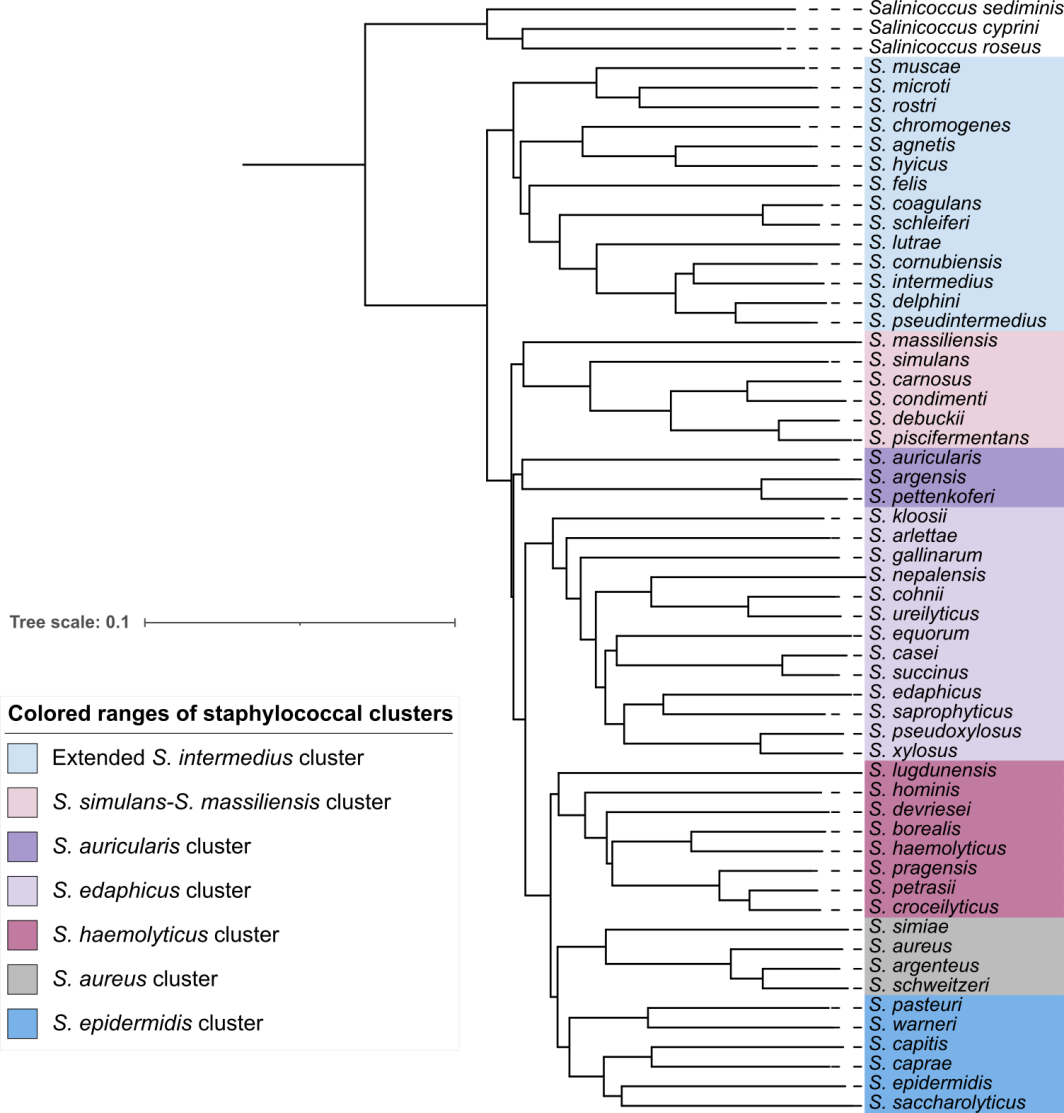
Neighbor-joining tree of the genus *Staphylococcus*. The tree, based on Mash genome distances, was constructed using Mashtree v1.2.0 with default parameters.

A neighbor-joining tree based on Mash genome distances was constructed using three *Salinicoccus* strains as an outgroup to root the tree. Contrary to the 15 clusters defined by Lamers et al. (29), our analysis divided the *Staphylococcus* genus into seven clusters (Fig. 1).

### Fibronectin binding and internalization into human osteoblasts

**Fig 2:**
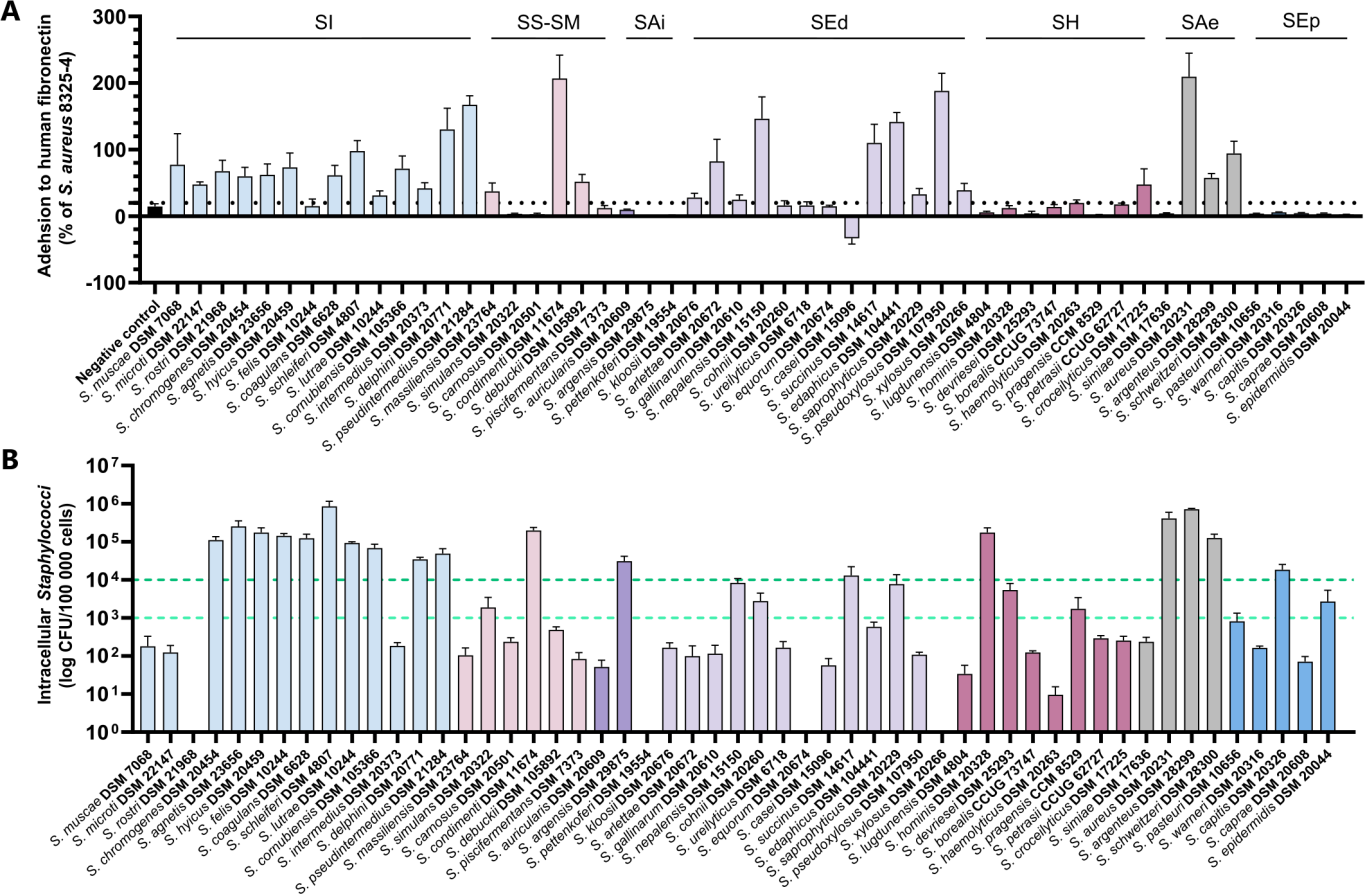
Assessment of human fibronectin adhesion and internalization into human osteoblasts for *Staphylococcus* spp. Experiments were performed independently at least three times. Results are presented as the mean ± standard error of the mean. (**A**) *In vitro* fibronectin adhesion capacity of 53 reference strains of staphylococci validly published until 2020. The data are expressed as a percentage of the adhesion ability relative to the *S. aureus* 8325-4 strain (positive control). The *S. aureus* DU5883 strain, which carries mutations in the *fnbA* and *fnbB* genes encoding the two isoforms FnBPA and FnBPB, was used as a negative control. (**B**) Quantification of staphylococcal internalization into MG-63 human osteoblasts. The thresholds defining low and high internalization capacities are 10^3^ and 10^4^ intracellular staphylococci per 10^5^ infected cells, respectively. SAe, *S. aureus* cluster; SAi, *S. auricularis* cluster; SEd, *S. edaphicus* cluster; SEp, *S. epidermidis* cluster; SH, *S. haemolyticus* cluster; SI, extended *S. intermedius* cluster; SS-SM, *S. simulans–S. massiliensis* cluster.

Fibronectin adhesion was assessed for all reference strains, with adhesion higher than the negative control considered as evidence of binding activity (Fig. 2A). Strains from the *S. auricularis*, *S. epidermidis*, and *S. haemolyticus* clusters showed minimal adhesion (<19.7% compared to the positive control), except *S. croceilyticus* (47.5%). In contrast, strains from the *S. aureus, S. edaphicus,* and extended *S. intermedius* clusters exhibited moderate to strong adhesion (24.8–209.5%). Within the *S. simulans-S. massiliensis* cluster, adhesion varied considerably, with *S. condimenti* showing high adhesion (206.8%), while *S. carnosus*, phylogenetically closest to *S. condimenti*, displayed no adhesion (Fig. 2A).

The internalization capacity of all reference strains was evaluated using MG-63 osteoblasts (Fig. 2B). Low internalization was defined as <10^3^ intracellular bacteria per 10^5^ infected cells, while high internalization was set at >10^4^ bacteria per 10^5^ cells. Within the *S. aureus* cluster, *S. aureus*, *S. argenteus*, and *S. schweitzeri* exhibited strong internalization, whereas *S. simiae* showed weak internalization. Similarly, most species within the extended *S. intermedius* cluster demonstrated high internalization, except *S. muscae*, *S. microti*, and *S. rostri*, which displayed weak or negligible invasion (Fig. 2B). In other clusters, most strains lacked internalization capacity, but *S. condimenti*, *S. argensis*, *S. succinus*, *S. hominis*, and *S. capitis* were highly internalized (2.0 × 10^5^, 3.1 × 10^4^, 1.3 × 10^4^, 1.7 × 10^5^, and 1.8 × 10^4^ bacteria per 10^5^ infected cells, respectively). Additionally, *S. simulans*, *S. nepalensis*, *S. cohnii*, *S. saprophyticus*, *S. devriesei*, *S. pragensis*, and *S. epidermidis* showed moderate internalization (1.7 × 10^3^ to 8.4 × 10^3^ intracellular bacteria per 10^5^ infected cells) (Fig. 2B). Fibronectin adhesion and osteoblast internalization varied widely among species, with no direct correlation observed between the two.

### *In silico* identification of FnBP homologs in internalized *Staphylococcus* strains

*In silico* analyses were conducted to determine whether internalization of species (*n* = 27) could be supported by the presence of FnBP homologs. Sequence homology between FnBPA of *S. aureus* and protein sequences from genomes of *Staphylococcus* strains with internalization levels above 10^3^ intracellular bacteria per 10^5^ infected cells identified 33 candidates. FnBPA is defined by four main domains: YSIRK Gram-positive signal peptide, SDR-like Ig domain, fibrinogen-binding domain 2, and LPXTG cell wall anchor domain. Three candidates lacking these domains and four containing serine-aspartate (SD) repeats typical of Sdr proteins were excluded. To investigate the position of the remaining 26 candidate homologs in their respective genomes, the genetic environment of each of these genes was then analyzed to determine their similarity (Table S1). A “genetic environment” was defined by five genes upstream and five downstream of the FnBP-like protein. Environments were classified as non-similar if no reference genes were present, if identified genes were not in the same order, or if the organization was different. A systematic search across *Staphylococcus* genomes identified six additional potential candidates, though three were removed for lacking FnBP-specific domains. Ultimately, 29 FnBP-like proteins were identified and named with “S” for *Staphylococcus*, two letters from the species name, and “s” for surface protein (Table S2).

**Fig 3:**
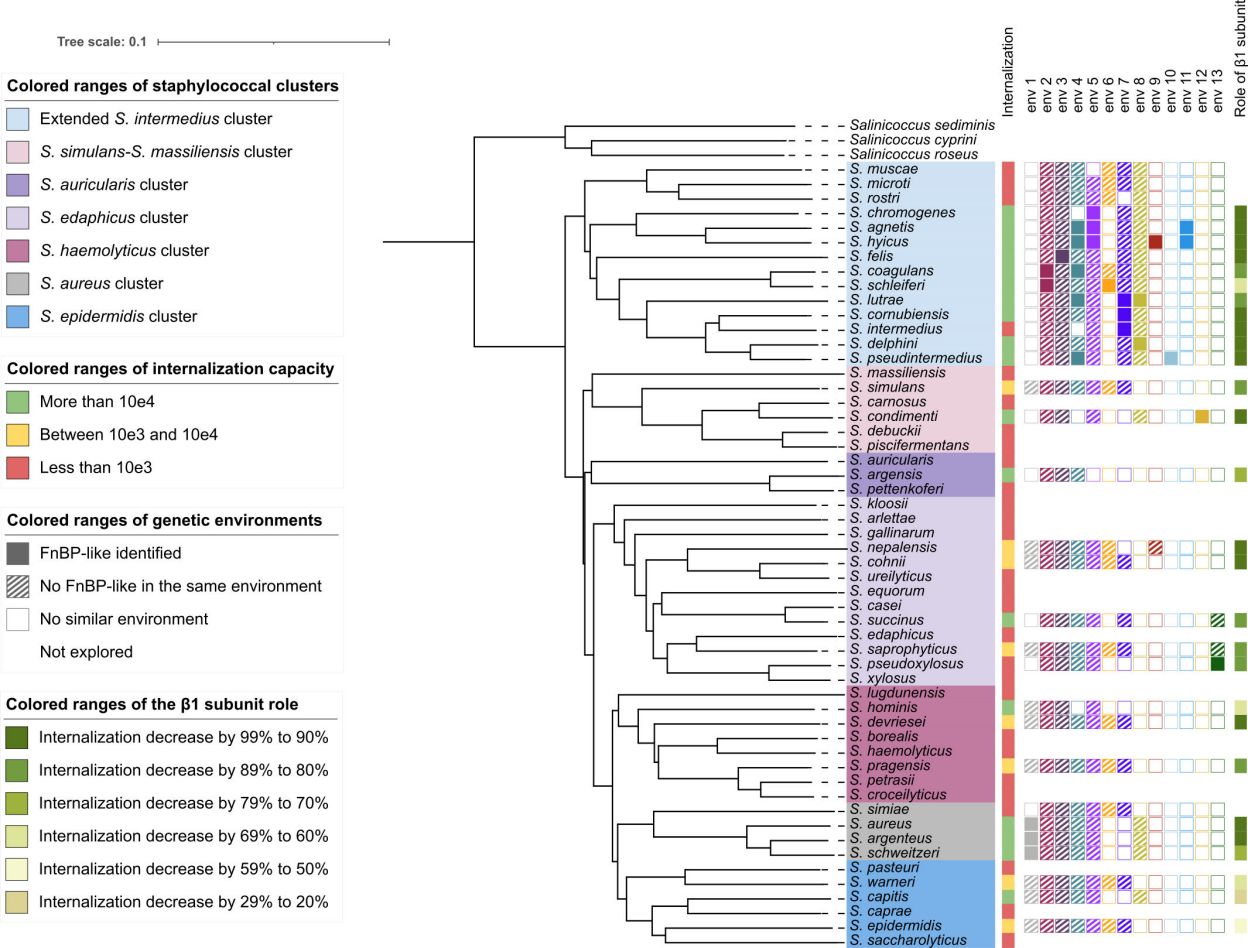
Neighbor-joining tree of the genus *Staphylococcus* and identification of FnBP homologous proteins. The tree, based on Mash genome distances, was constructed using Mashtree v1.2.0 with default parameters. The internalization capacity, the presence of FnBP homologous proteins and their genetic environment, as well as the role of the β1 subunit of the α5β1 integrin in the internalization pathway of each species, are illustrated. The internalization capacity of *Staphylococcus* species was evaluated using MG-63 human osteoblasts, as previously described (26). The “genetic environments” consist of 10 genes: five upstream and five downstream of the FnBP homologous protein. An environment is considered dissimilar if genes from the reference environment are absent, or if they are identified but not in the same order or not organized together in the genome. The impact of β1 subunit deletion in the α5β1 integrin is measured by the reduction in the proportion of *Staphylococcus* internalization in murine osteoblasts lacking the β1 subunit (OB-β1^-−/−^) compared to wild-type murine osteoblasts (OB-β1^+/+^).

FnBP-like genes were identified in almost all *Staphylococcus* strains with high internalization (Fig. 3). In the *S. aureus* cluster, two FnBP-like proteins were identified within a conserved genetic environment in *S. aureus* (FnBPA and FnBPB), *S. argenteus* (SarsA and SarsB), and *S. schweitzeri* (SswsA and SswsB) (Fig. 3; Table S1, environment 1). Evolutionary tree based on the A domain (Fig. S2A) or full protein sequences (Fig. S2B) showed that FnBPA, SarsA, and SswsA grouped together as well as FnBPB, SarsB, and SswsB. In *S. simiae*, which lacks internalization ability, no FnBP-like protein was identified in a similar environment or elsewhere in the genome.

In the extended *S. intermedius* cluster, which concentrates most of the high internalization species, up to four FnBP-like proteins were found in the different genomes. Four FnBP-like genes were identified in *S. hyicus*, whereas three were identified in *S. agnetis* and *S. lutrae*. Some of these FnBP-like proteins found in different species were grouped in the evolutionary trees (Fig. S2A and B) and were found in a similar genetic environment, such as SslsA from *S. schleiferi* and ScasA from *S. coagulans* in environment 2 or SchsA from *S. chromogenes*, SagsB from *S. agnetis,* and ShysB from *S. hyicus* in environment 5 (Fig. 3; Table S1).

Interestingly, an FnBP-like protein (SpxsA) was also identified in *S. pseudoxylosus*, despite its inability to internalize into osteoblasts. However, *S. pseudoxylosus* exhibits strong fibronectin adhesion, suggesting its FnBP-like protein contributes to adhesion but not internalization. In contrast, no FnBP-like proteins were found in *S. argensis*, *S. succinus*, *S. hominis*, and *S. capitis*, though these species demonstrate high internalization rates (1.2 × 10^4^ to 1.7 × 10^5^ intracellular bacteria per 10^5^ infected cells, Fig. 2B and 3). Similarly, no FnBP-like proteins were identified in strains exhibiting moderate internalization capacity (10^3^–10^4^ intracellular bacteria per 10⁵ infected cells, Fig. 3).

High internalization capacity mostly correlated with the presence of FnBP-like proteins. The distribution of proteins does not match species phylogeny but rather genetic environments (Fig. 1 and 3; Fig. S2; Table S1), reinforcing the hypothesis of multiple independent acquisitions across *Staphylococcus* species and genus.

### Role of the β1 subunit in the internalization pathway into osteoblasts

**Fig 4:**
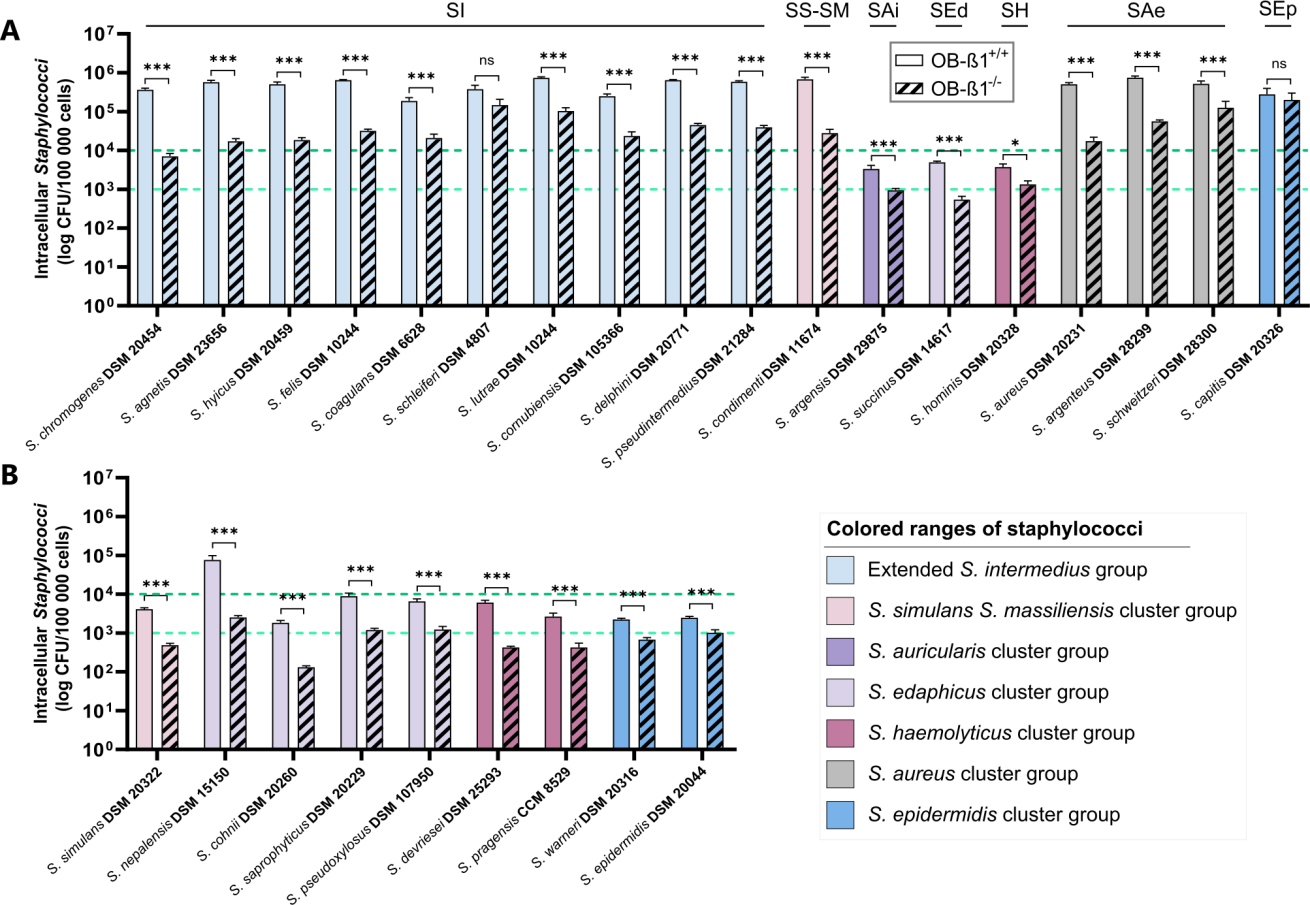
Characterization of the role of the β1 subunit of the α5β1 integrin in the internalization pathway. The role of the β1 subunit of the α5β1 integrin was investigated using OB-β1^-/-^ murine osteoblasts, which lack the β1 subunit of the α5β1 integrin, and wild-type murine osteoblasts (OB-β1^+/+^). Experiments were performed independently at least three times. Results are presented as the mean ± standard error of the mean. (**A**) Characterization of the role of the β1 subunit of the α5β1 integrin in the internalization pathway of *Staphylococcus* species with a high internalization capacity in MG-63 human osteoblasts. (**B**) Characterization of the role of the β1 subunit of the α5β1 integrin in the internalization pathway of *Staphylococcus* species with an average internalization capacity in MG-63 human osteoblasts. Mann-Whitney test: *P* value non-significant (ns), 0.01 (*), 0.001 (**), and 0.0001 (***). SAe, *S. aureus* cluster; SAi, *S. auricularis* cluster; SEd, *S. edaphicus* cluster; SEp, *S. epidermidis* cluster; SH, *S. haemolyticus* cluster; SI, extended *S. intermedius* cluster; SS-SM, *S. simulans–S. massiliensis* cluster.

The impact of the β1 subunit of the α5β1 integrin on *Staphylococcus* internalization was assessed in species with an internalization rate above 10⁴ intracellular bacteria per 10^5^ infected cells (Fig. 4A). The internalization capacity of *S. aureus* decreased by 96.6% in OB-β1^−/−^ osteoblasts compared to OB-β1^+/+^ cells. Sixteen *Staphylococcus* species exhibited a significant reduction (64.1% for *S. hominis* to 98.1% for *S. chromogenes*), while *S. schleiferi* and *S. capitis* remained unaffected. A FnBP-like protein was present in all species except *S. capitis*.

Species with moderate internalization (10^3^–10^4^ bacteria per 10^5^ cells) and two species near the threshold (*S. pseudoxylosus* and *S. warneri*, Fig. 4B) were also examined. Internalization declined significantly (59.0% for *S. epidermidis* to 96.7% for *S. nepalensis*). No FnBP-like proteins were found in these strains except *S. pseudoxylosus*, though its SpxA repeat region lacked Fn-binding domains (Fig. S1). These results highlight the essential role of the β1 subunit in *Staphylococcus* osteoblast internalization.

### Reduced internalization due to truncated FnBP-like protein

**Fig 5:**
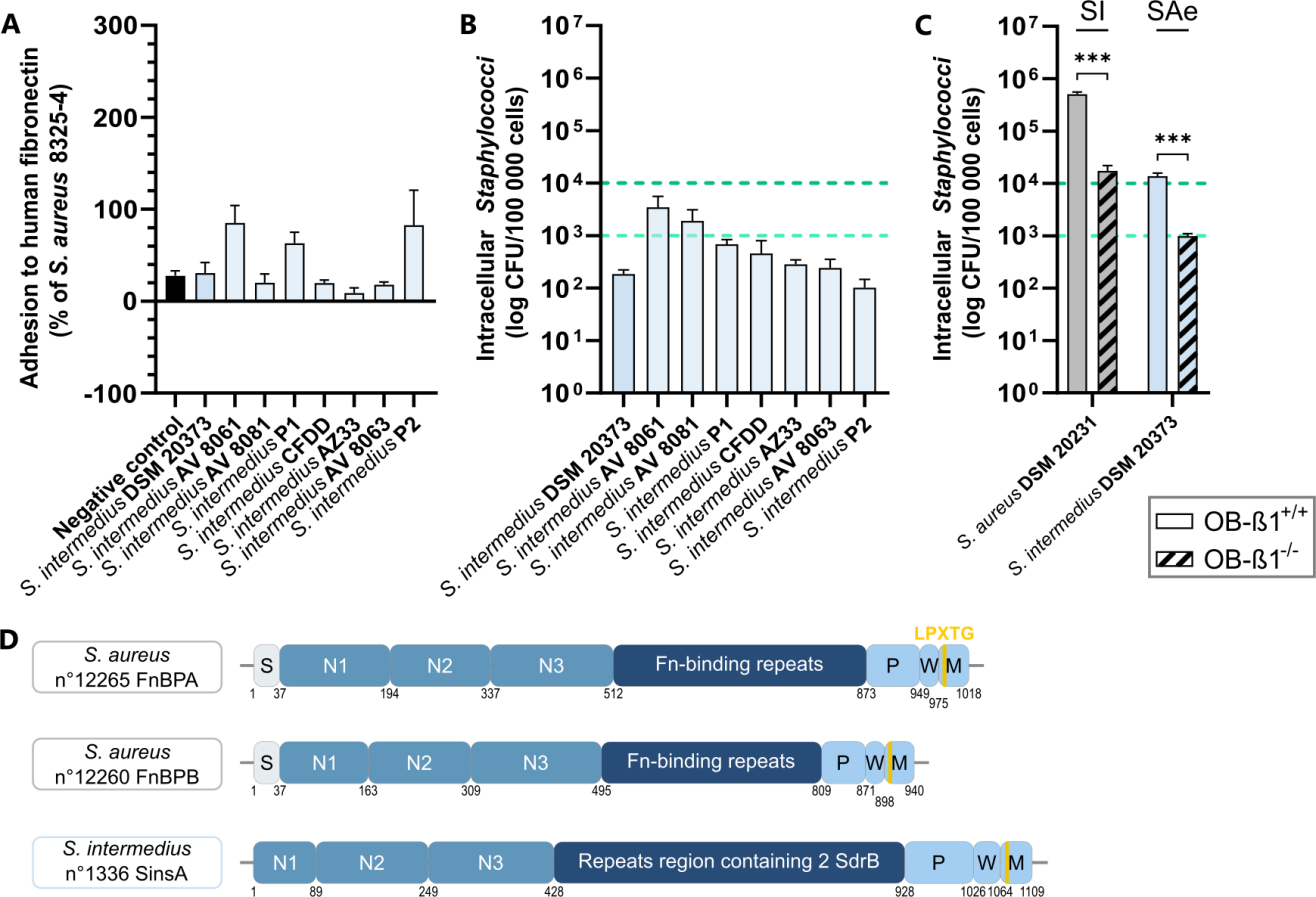
Characterization of the internalization pathway of *Staphylococcus intermedius*. Experiments were performed independently at least three times. Results are presented as the mean ± standard error of the mean. (**A**) Evaluation of *S. intermedius* adhesion to fibronectin. The data are expressed as a percentage of the adhesion ability relative to the positive control strain *S. aureus* 8325-4. The negative control strain corresponds to the fibronectin adhesion capacity of *S. aureus* DU5883, which is deleted for the *fnbA* and *fnbB* genes encoding the two isoforms FnBPA and FnBPB. (**B**) Quantification of *S. intermedius* internalization into MG-63 human osteoblasts. (**C**) Characterization of the role of the β1 subunit of the α5β1 integrin in the internalization pathway of *S. intermedius*. The role of the β1 subunit of the α5β1 integrin was investigated using OB-β1^−/−^ murine osteoblasts, which lack the β1 subunit of the α5β1 integrin, and wild-type murine osteoblasts (OB-β1^+/+^). SAe, *S. aureus* cluster; SI extended, *S. intermedius* cluster. Mann-Whitney test: *P* value non-significant (ns), 0.01 (*), 0.001 (**), and 0.0001 (***). (**D**) Domain organization of *S. intermedius* FnBP homologous protein. S, signal peptide. N1, N2, and N3 represent the A domain. Fn-binding repeats, fibronectin-binding protein; M, membrane-spanning domain; P, proline-rich repeats; W, wall-spanning domain. The protein numbers correspond to the sequences identified in the *in silico* analysis.

The reference strain *S. intermedius* DSM 20373 exhibited atypical behavior within the extended *S. intermedius* cluster, as it failed to internalize into human osteoblasts despite possessing an FnBP-like gene. Fibronectin adhesion assays of eight *S*. *intermedius* strains showed high variability, with adhesion rates ranging from 8.91% (below the negative control *S. aureus* DU5883) to 84.9% relative to the positive control *S. aureus* 8325-4 (Fig. 5A). Internalization capacity varied from 1.0 × 10^2^ to 3.5 × 10^3^ intracellular bacteria per 10^5^ infected cells (Fig. 5B), with no correlation between fibronectin adhesion and osteoblast internalization.

For *S. intermedius* DSM 20373, internalization decreased by 92.8%, indicating residual internalization is dependent on α5β1 integrin (Fig. 5C). *In silico* analysis identified an FnBP homolog, *S. intermedius* surface protein SinsA (Fig. 5D), composed of an A domain with three subdomains (N1, N2, and N3), a repeat region containing two SdrB domains, and an LPXTG motif at the C-terminal end. Unlike *S. aureus* FnBPA/B, SinsA lacks a signal peptide, which, along with its SdrB domains, may impact *S. intermedius* osteoblast internalization.

## DISCUSSION

The internalization of *S. aureus* into non-professional phagocytic cells is a key factor in the persistence of chronic infections (30). However, little is known about the internalization capacity of other *Staphylococcus* species involved in human infections. To address this gap, we evaluated staphylococcal internalization into NPPCs at the genus level. Given the frequent implication of staphylococci in BJIs, osteoblasts were selected as the host cells of interest.

Our findings reveal that staphylococcal internalization into osteoblasts is species-dependent and that the FnBP-fibronectin-α5β1 integrin pathway, initially described for *S. aureus*, is also utilized by most species capable of osteoblast internalization. Most species with this capacity belong to two genetic clusters: the *S. aureus* cluster and the extended *S. intermedius* cluster (Fig. 1).

The *S. aureus* cluster includes *S. aureus, S. argenteus, S. schweitzeri,* and *S. simiae*. While *S. aureus*, *S. argenteus*, and *S. schweitzeri* exhibit internalization capacity, *S. simiae* does not. The first three species possess two FnBP or FnBP-like isoforms encoded by contiguous genes within a conserved genetic environment. FnBPA, SarsA, and SswsA have high homology and seem to have a common ancestor. Similar observations can be made for FnBPB, SarsB, and SswsB (Fig. S2). In contrast, *S. simiae* lacks FnBP-like genes despite sharing the same genetic environment. Phylogenetic analyses place *S. simiae* on a distinct branch, supporting the hypothesis that internalization capacity was acquired by a common ancestor of *S. aureus*, *S. argenteus*, and *S. schweitzeri* after diverging from *S. simiae*. This hypothesis aligns with Suzuki et al., who suggested that *S. aureus* acquired virulence factors via horizontal gene transfer following its split from *S. simiae* (31). Notably, no published study has investigated *S. simiae* internalization in any host cell. Regarding *S. schweitzeri*, our findings corroborate Grossmann et al., who reported an internalization capacity similar to *S. aureus* in Vero cells across 58 strains of *S. schweitzeri*. Its ability to internalize raises concerns about its potential emergence as a zoonotic pathogen (32).

The extended *S. intermedius* cluster consists of 14 species, most originally isolated from animals (4). Among these, 10 demonstrated significant osteoblast internalization (Fig. 1). Species lacking internalization, *S. muscae*, *S. microti*, and *S. rostri*, also lack FnBP-like proteins. Similar to *S. simiae*, these species group together on an early-diverging phylogenetic branch, supporting the hypothesis that osteoblast internalization genes were acquired after their divergence. However, this cluster exhibits more genetic complexity than the *S. aureus* cluster. FnBP-like proteins in the 10 internalizing species were identified in diverse genetic environments. Some FnBP-like proteins are shared among closely related species, such as SchsA, SagsB, and ShysB in *S. chromogenes, S. agnetis*, and *S. hyicus*, all found in the same genetic environment. Additionally, the phylogenetic tree of FnBP-like proteins shows similarities among those located in environments 2, 3, 4, 9, and 11 (Fig. S2), confirmed by their functional domain organization (Fig. S1). The distribution of FnBP-like proteins across distinct genetic backgrounds, coupled with their frequent association with virulence factors, regulators, metal-related elements, and mobile genetic elements (Table S1, sheet “Environment type”), suggests horizontal gene transfer as a major contributor to their dissemination. Investigating insertion sites within these genetic environments may yield further insights.

While many species within the extended *S. intermedius* group are primarily associated with animals (4), our findings indicate that several possess the capacity to internalize into human osteoblasts. Importantly, human infections caused by species of the extended *S. intermedius* group have been reported even in immunocompetent hosts. For example, *S. pseudintermedius* has been implicated in endocarditis or prosthetic joint infections following close contact with dogs (9), *S. delphini* has been isolated in rare human cases (33). These observations suggest that internalization may represent an ancestral or functionally retained trait and be conserved across the genus, contributing to host colonization and opportunistic infection, rather than being restricted to classical pathogens such as *S. aureus*. This highlights the potential clinical relevance of non-aureus staphylococci and supports further investigation into their virulence mechanisms.

While *S. intermedius* exhibits limited internalization despite possessing an FnBP-like protein (SinsA), the absence of a signal peptide in SinsA may explain this phenotype. However, because *S. delphini*’s SdesA protein displays similar characteristics, this assumption remains uncertain. Furthermore, the SdsY protein previously reported by Maali et al. (26) was not detected in our analysis.

Two FnBP-like proteins, ScnsA and SpxsA, were identified in *S. condimenti* and *S. pseudoxylosus*, respectively, two species outside the *S. aureus* and extended *S. intermedius* clusters. Within their respective clusters, they are the only species possessing an FnBP-like protein. *S. condimenti* exhibited strong internalization capacity in osteoblasts, with ScnsA found in a unique genetic environment. Phylogenetic analysis showed that ScnsA is related to SchsA, SagsB, and ShysB, FnBP-like proteins in *S. chromogenes, S. agnetis,* and *S. hyicus* within genetic environment 5 (Fig. 1; Fig. S2). This suggests horizontal gene transfer from one of these species to *S. condimenti*, followed by integration into a distinct genetic environment.

A similar situation is observed for *S. pseudoxylosus*, except this species does not internalize into osteoblasts. Notably, SpxsA clustered within the distinct branch grouping proteins from environments 4, 6, 7, and 8, rather than following the main distribution observed for most proteins. This pattern suggests that SpxsA may have experienced a different evolutionary trajectory, possibly involving horizontal gene transfer into a distinct genetic environment. However, the repeated region of SpxsA contains an atypical keratinocyte proline-rich domain, lacking fibronectin-binding repeats, which may explain why *S. pseudoxylosus* is unable to internalize into osteoblasts.

Twelve other species showed moderate to strong osteoblast internalization. No FnBP-like proteins were identified in their genomes, nor in analyses of genetic environments. These findings align with their lack of fibronectin adhesion, except for *S. cohnii*. However, their internalization appears largely dependent on α5β1 integrin. Other proteins, such as Atl, may mediate non-*S*. *aureus* staphylococcal uptake via α5β1 integrin or Hsc70 (34).

Interestingly, several species, including *S. argensis*, *S. hominis*, and *S. felis*, exhibited significant osteoblast internalization despite the absence of FnBP-like protein in their genomes. This inconsistency between *in silico* predictions and experimental data suggests that alternative, FnBP-independent pathways contribute to staphylococcal uptake. Previous studies have demonstrated that proteins such as Atl can mediate internalization via the α5β1 integrin pathway (34, 35). Therefore, a similar β1-integrin-dependent, FnBP-independent mechanism may exist in these species. These findings highlight the limitations of current *in silico* analyses in predicting all fibronectin-binding proteins and emphasize the need for experimental validation to fully elucidate the molecular determinants of internalization across the *Staphylococcus* genus.

In this context, the identification of FnBP-like proteins in other *Staphylococcus* species further illustrates the diversity of surface proteins potentially involved in host cell interaction. Proteins SpisA and SpisB are two FnBP-like proteins identified following the method we described. According to Bannoehr et al., the two proteins SpsD and SpsL identified in *S. pseudintermedius* ED999 are also present in the *S. pseudintermedius* type strain genome (36). The SpsL sequence shares 97.43% homology with SpisA according to default parameters of BlastP. However, SpisB presents only 38.52% and 24.34% homology with SpsD and SpsL, respectively, suggesting the identification of a novel putative surface protein.

Our study is not the first to analyze virulence factors at the genus level. Pickering et al. (37) examined plasma clotting capacity linked to the *vwb* gene encoding von Willebrand factor-binding protein (vWbp) (37). Notably, all strains capable of clotting plasma in their study also exhibited high osteoblast internalization, except *S. intermedius*. The authors proposed that this phenotype evolved as an adaptation to the host [56]. FnBP-like proteins, found in multiple clusters within the *Staphylococcus* genus, align with species capable of internalization. Similar to vWbp, FnBP-like proteins could represent key virulence factors in host-pathogen interactions.

Several limitations should be considered. The primary limitation is that only reference strains of each species were examined, making broad conclusions about species-wide internalization capacity uncertain. *S. aureus* is known to exhibit strain-dependent internalization variation (38, 39), as illustrated in our study by *S. intermedius*, whose internalization fluctuated by more than one log depending on the strain. Future studies should analyze multiple strains per species to account for intraspecies diversity.

Additionally, we focused solely on osteoblast internalization, using the MG-63 cell line. While primary osteoblasts might provide a more physiopathologically relevant model, their cultivation is complex, making large-scale infections challenging. Testing staphylococcal internalization in alternative cell models, such as keratinocytes or endothelial cells, would help assess host cell specificity. The classification of species based on internalization capacity likely varies by host cell type. Strobel et al. found that *S. aureus* internalization varies depending on cell type, primary versus cell line conditions, and cytotoxicity (38). Our observed differences in *S. intermedius* internalization between OB-β1^+/+^ murine osteoblasts and MG-63 human osteoblasts further underscore the potential impact of host origin.

Also, the use of arbitrary thresholds to classify invasion levels represents a limitation of this study. Nevertheless, such thresholds were necessary to enable consistent comparison across isolates, and the overall trends observed remain robust. In addition, while this study provides a comprehensive genus-wide overview of FnBP-like proteins and internalization phenotypes, it remains descriptive. Future studies investigating the functional contribution of these FnBP-like proteins, for instance through gene deletion mutants, would be valuable to establish a direct link between genotype and phenotypes.

The definition of genetic environments in our analysis of FnBP-like proteins presents certain limitations. These environments were arbitrarily defined based on 10 genes flanking the FnBP-like protein. Since additional genes may have been acquired alongside FnBP-like proteins, shifts in the original environment could lead to misinterpretations.

Another key limitation is the classification of FnBP-like proteins. Among the identified proteins, eight contain at least one SdrB domain, which, according to Foster, is characteristic of Clf, Cna, and Sdr proteins within the MSCRAMMs family (40). Specifically, Cna has four B repeats, while Sdr and ClfA contain two (41). This suggests that the FnBP-like proteins *ScasB*, *SslsB*, *SlusB*, *ScrsA*, *SinsA*, *SdesA*, and *SpisA*, identified in *S. coagulans*, *S. schleiferi*, *S. lutrae*, *S. cornubiensis*, *S. intermedius*, *S. delphini*, and *S. pseudintermedius*, may belong to the Clf, Cna, or Sdr families. Studies by Josefsson et al. and Patti et al. identified Cna and ClfA as virulence determinants in staphylococcal BJIs (42, 43), suggesting further investigation into their role in the α5β1 integrin-dependent internalization pathway. Given the overlap in protein features, the current classification may be overly rigid. Further research is required to refine protein categorization or establish new classifications.

This study examined bacterial internalization in the *Staphylococcus* genus using osteoblasts as a model. Internalization varied across species, with half of the 53 tested species exhibiting high osteoblast internalization. The *S. aureus* “FnBP-fibronectin-α5β1 integrin” pathway is conserved in 27 species. *In silico* analyses identified 29 FnBP-like proteins, suggesting multiple acquisitions and/or horizontal gene transfer events throughout *Staphylococcus* evolution.

## MATERIALS AND METHODS

### Phylogenetic analysis

Genomes in FASTA format were downloaded from NCBI (Table S3) and annotated using Bakta v1.6.1 with default parameters. A neighbor-joining tree based on Mash genome distances was constructed using Mashtree v1.2.0 with default parameters. Three *Salinicoccus* strains (*S. sediminis* SV-16, *S. cyprini* CT19, and *S. roseus* W12) were used as an outgroup to root the tree.

### Bacterial strains

A collection of 53 reference strains of validly published staphylococci (as of 2020) and gentamicin-sensitive was used, each representing a distinct *Staphylococcus* species (Table S3, sheet “Staphylococcal strains”). *S. aureus* 8325-4 was used as a positive control, given its ability to adhere to fibronectin-coated surfaces (44). The isogenic mutant *S. aureus* DU5883, which lacks FnBPs, served as a negative control for fibronectin adhesion assays (44).

### Cell culture

Three osteoblast cell lines were cultured: the human osteoblastic MG-63 cell line and two murine osteoblastic cell lines (45, 46). The murine cell lines were derived from the calvaria of transgenic mice: OB-β1^+/+^, which expresses a functional β1 integrin subunit, and OB-β1^−/^−, which lacks the *itgb1* gene encoding β1 integrin (46). Cells were maintained in 250 mL flasks with Dulbecco’s modified Eagle medium containing phenol red, supplemented with 10% heat-inactivated fetal bovine serum and 100 µg/mL penicillin/streptomycin. Cells were incubated for 1 week at 37°C with 5% CO_2_ before plating.

### Bacterial culture

Bacteria were grown in brain-heart infusion broth for 18 h at 37°C with shaking at 180 rpm to reach stationary phase. Cultures were centrifuged, and the pellet was resuspended in phenol red-free cell culture medium. Bacterial suspensions were plated on tryptone soy agar (TSA) to determine concentrations after overnight incubation at 37°C. Suspensions were stored at 4°C until infection.

### Fibronectin adhesion assays

Fibronectin adhesion assays were performed in 96-well flat-bottom microplates coated with 200 µL of either 50 µg/mL Corning human fibronectin or 1% bovine serum albumin (BSA) diluted in phosphate-buffered saline (PBS), followed by incubation at 4°C for 18 h with continuous stirring (50 rpm). Wells were washed three times with PBS/BSA for 20 min at 37°C. BacLight RedoxSensor Green Vitality reagent (Life Technologies) was added to bacterial suspensions at 10^8^ CFU/mL to reach a final concentration of 1 µM. Suspensions were incubated in the dark for 15 min at 37°C with continuous shaking (80 rpm). Each bacterial strain (100 µL) was added to fibronectin-coated wells and incubated for 45 min at 37°C with shaking (80 rpm). Wells were washed three times with PBS, and fluorescence was measured using an Infinite M Nano+ (Tecan) plate reader. Results are expressed as the percentage fluorescence relative to the *S. aureus* 8325-4 positive control, after subtracting non-specific interactions measured with BSA.

### Determination of staphylococcal internalization capacity

Cells were seeded at a density of 100,000 cells per well and incubated for 48 h at 37°C with 5% CO^2^ in a 24-well tissue culture plate. Wells were washed two times with PBS, and cells were infected with 1 mL bacterial suspension (MOI = 100 bacteria per cell). The number of cells was counted on the day of infection to verify MOI. Plates were incubated for 2 h at 37°C with 5% CO_2_. After incubation, wells were washed once with PBS, and 1 mL of 20 µg/mL gentamicin was added. Plates were incubated for 1 h at 37°C with 5% CO_2_. After washing with 700 µL PBS, cells were lysed with 1 mL sterile water and incubated for 30 min at 37°C with 5% CO_2_. Lysates were plated on TSA and incubated at 37°C for 24 h.

### Role of β1 integrin in highly internalized staphylococci

The same method used for determining internalization capacity was applied, except OB-β1^+/+^ and OB-β1^−/−^ osteoblasts were plated together in the same 24-well plate to facilitate direct comparison (46).

### Identification of *fnb* gene homologs and genomic environment analysis

BLASTp v2.13.0+ was used to identify *fnb*-like sequences in all genomes. The query sequence was the FnBPA reference protein from UniProt (ID P14738). Sequences with ≥25% identity and ≥40% coverage were retained. InterProScan v92.0 or higher was used to analyze protein domains, comparing candidate proteins to the reference sequence. FnBPA consists of four main domains: YSIRK Gram-positive signal peptide (IPR005877), SDR-like Ig domain (IPR041171), fibrinogen-binding domain 2 (IPR011266), and LPXTG cell wall anchor domain (IPR019931). Proteins containing serine-aspartate (SD) repeats, characteristic of Sdr proteins, were excluded (47). Fibronectin-binding repeats were not used to search for homologous candidates because of their high variability (19). The schematic representation of each protein sequence was generated using InterPro domain search and SignalP 5.0 for signal peptide prediction. Genomic environments of candidates were manually examined to identify additional FnBP-like proteins by comparing genetic regions. Environments were defined as 10 adjacent genes—five upstream and five downstream of the *fnb* homologous protein (Table S1).

### Molecular phylogenetic analysis

Sequences of 29 homologous proteins were aligned using MAFFT v7 with default parameters. A neighbor-joining tree was constructed based on this alignment, excluding gap-containing sequences. The Jones-Taylor-Thornton sequence evolution model was applied, with estimated sequence heterogeneity (48).

## SUPPLEMENTAL MATERIAL

Figure S1 mbio.01697-25-s0001.tiff Organization of the protein structure of FnBP-like proteins.

Figure S2 mbio.01697-25-s0002.tiff Phylogenetic tree of FnBP-like proteins.

Legends mbio.01697-25-s0003.docx Legends for the supplemental figures and tables.

Table S1 mbio.01697-25-s0004.xlsx Genomic environment of FnBP-like protein-coding genes.

Table S2 mbio.01697-25-s0005.xlsx FnBP-like proteins and previous candidates.

Table S3 mbio.01697-25-s0006.xlsx List of strains.

ASM does not own the copyrights to Supplemental Material that may be linked to, or accessed through, an article. The authors have granted ASM a non-exclusive, world-wide license to publish the Supplemental Material files. Please contact the corresponding author directly for reuse.
