# Supplementary figures and images for: Staphylococcal internalization into osteoblasts: a partially conserved mechanism across the genus
Source: mBio. 2025 Dec 16;17(1):e01697-25. doi: 10.1128/mbio.01697-25 (PMC12802258; doi:10.1128/mbio.01697-25)

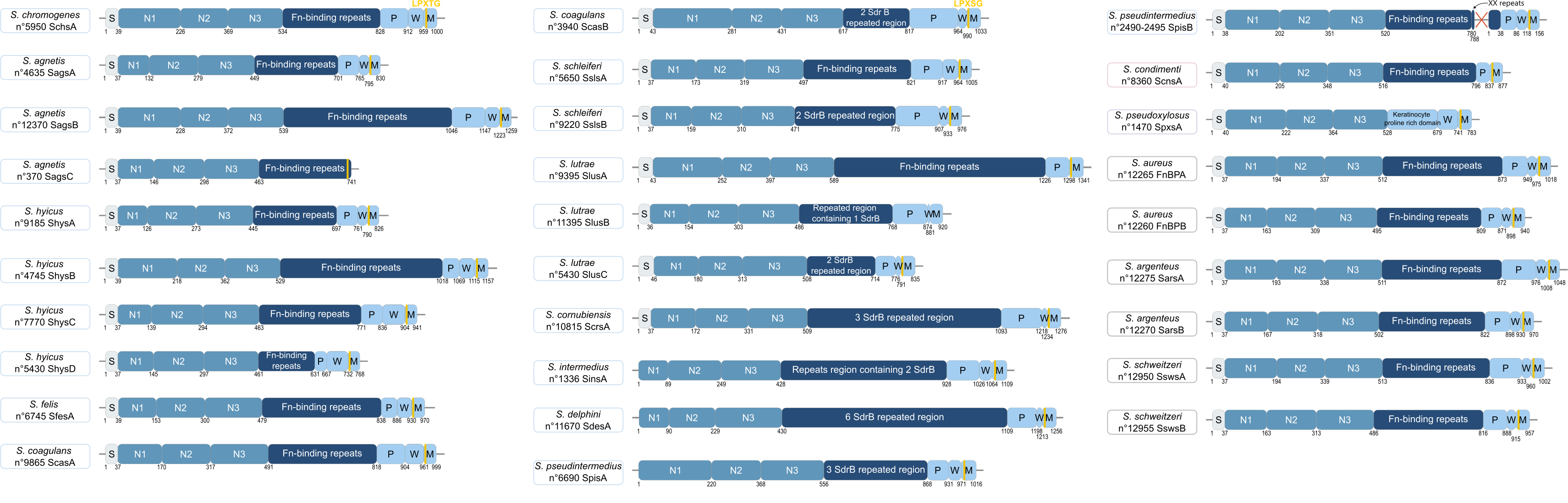

Supplement: Figure S1 — Organization of the protein structure of FnBP-like proteins. [file mbio.01697-25-s0001.tiff]

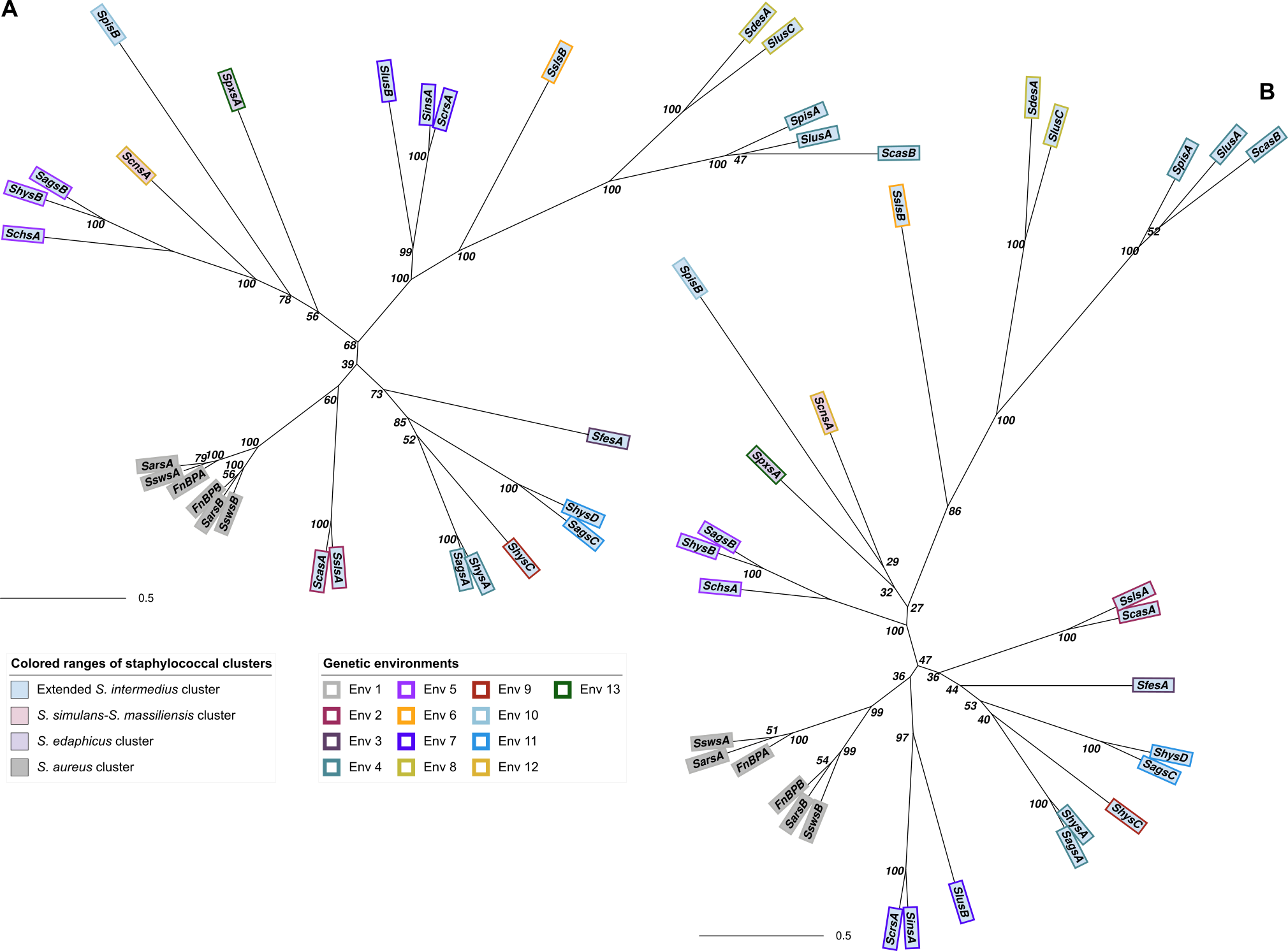

Supplement: Figure S2 — Phylogenetic tree of FnBP-like proteins. [file mbio.01697-25-s0002.tiff]
