# Supplementary material for: Staphylococcal internalization into osteoblasts: a partially conserved mechanism across the genus
Source: mBio. 2025 Dec 16;17(1):e01697-25. doi: 10.1128/mbio.01697-25 (PMC12802258; doi:10.1128/mbio.01697-25)
Supplement: Legends — for the supplemental figures and tables. [file mbio.01697-25-s0003.docx]

Supplementary legend

**Supplementary Figure 1: Domain organization of the FnBP-like proteins.** The signal peptide (S) is followed by the A domain (N1, N2, and N3) of the repeat sequence region, the proline-rich domain (P), and finally the wall-spanning (W) and membrane (M) domains. The M domain contains the Leu–Pro–X–Thr–Gly (LPXTG) motif. This representation was constructed based on domain recognition performed using the InterPro software.

**Supplementary Figure 2: Molecular phylogenetic trees of the 29 proteins homologous to FnBP.** Amino acid sequences were aligned using MAFFT software, version 7, with default parameters. Phylogenetic trees were generated using the IQ-TREE Los Alamos Lab web server, applying a maximum-likelihood approach with standard bootstrap support (46). Proteins are colored according to the group to which the species from which they were identified belong. These proteins are framed according to the color of the genetic environments in which they are present. **A:** Molecular phylogenetic tree of the complete 29 proteins homologous to FnBP. **B:** Molecular phylogenetic tree of the A domains of the 29 proteins homologous to FnBP.

**Supplementary Table 1: Genomic environment of FnBP-like protein-coding genes.** The “Environment identification” sheet corresponds to the identification of all the genomic environments that contain at least one FnBP-like protein, as well as their occurrence across all genomes of species exhibiting high internalization into osteoblasts. The “Environment type” sheet highlights all the genomic environments surrounding an FnBP-like protein-coding gene, grouped by environment type. Genes involved in horizontal gene transfer and virulence are highlighted in color, with the legend provided in the “Color legend” sheet.

**Supplementary Table 2: FnBP-like proteins.** All the identified FnBP-like protein-coding genes are listed in the “FnBP-like” sheet, with the corresponding species and accession numbers. The “FnBP-like candidates” sheet corresponds to all the candidates that have been identified, indicating those that were not retained and the reasons for their exclusion.

**Supplementary Table 3: Collection of all the strains used in this study.** The “Staphylococcal strains” sheet lists all the Staphylococcus reference strains investigated and the three *Salinicoccus* species used to generate the phylogenetic tree. The “*S. intermedius* strains” sheet lists all *Staphylococcus intermedius* strains studied.
